# Screening of soy protein-derived hypotriglyceridemic di-peptides *in vitro *and *in vivo*

**DOI:** 10.1186/1476-511X-10-85

**Published:** 2011-05-22

**Authors:** Nao Inoue, Koji Nagao, Kotaro Sakata, Naomi Yamano, Pathma Elgoda Ranawakage Gunawardena, Seo-Young Han, Toshiro Matsui, Toshihiro Nakamori, Hitoshi Furuta, Kiyoharu Takamatsu, Teruyoshi Yanagita

**Affiliations:** 1 Department of Applied Biochemistry and Food Science, Saga University, Saga 840-8502, Japan; 2 Department of Bioscience and Biotechnology, Graduate School of Kyushu University, Fukuoka 812-8581, Japan; 3 Fuji Oil Co, Osaka 598-8540, Japan; 4 Graduate School of Agricultural Science, Tohoku University, Miyagi 981-8555, Japan

## Abstract

**Background:**

Soy protein and soy peptides have attracted considerable attention because of their potentially beneficial biological properties, including antihypertensive, anticarcinogenic, and hypolipidemic effects. Although soy protein isolate contains several bioactive peptides that have distinct physiological activities in lipid metabolism, it is not clear which peptide sequences are responsible for the triglyceride (TG)-lowering effects. In the present study, we investigated the effects of soy protein-derived peptides on lipid metabolism, especially TG metabolism, in HepG2 cells and obese Otsuka Long-Evans Tokushima fatty (OLETF) rats.

**Results:**

In the first experiment, we found that soy crude peptide (SCP)-LD3, which was prepared by hydrolyze of soy protein isolate with endo-type protease, showed hypolipidemic effects in HepG2 cells and OLETF rats. In the second experiment, we found that hydrophilic fraction, separated from SCP-LD3 with hydrophobic synthetic absorbent, revealed lipid-lowering effects in HepG2 cells and OLETF rats. In the third experiment, we found that Fraction-C (Frc-C) peptides, fractionated from hydrophilic peptides by gel permeation chromatography-high performance liquid chromatography, significantly reduced TG synthesis and apolipoprotein B (apoB) secretion in HepG2 cells. In the fourth experiment, we found that the fraction with 0.1% trifluoroacetic acid, isolated from Frc-C peptides by octadecylsilyl column chromatography, showed hypolipidemic effects in HepG2 cells. In the final experiment, we found that 3 di-peptides, Lys-Ala, Val-Lys, and Ser-Tyr, reduced TG synthesis, and Ser-Tyr additionally reduced apoB secretion in HepG2 cells.

**Conclusion:**

Novel active peptides with TG-lowering effects from soy protein have been isolated.

## Background

In industrialized countries, lifestyle-related diseases such as hyperlipidemia, arteriosclerosis, diabetes mellitus, and hypertension are widespread and increasingly prevalent, thus contributing to the increases in cardiovascular morbidity and mortality [1,2]. Accompanied by the rapid increase in the number of elderly people, this increase in lifestyle-related diseases becomes important not only medically, but also socioeconomically. A clustering of metabolic disorders in an individual, defined as metabolic syndrome, is known to increase cardiovascular morbidity and mortality. Although the pathogenesis of metabolic syndrome is complicated and the precise details of its underlying mechanisms are not known, lipid abnormality is now proposed as a feature of metabolic syndrome along with insulin resistance [1-3]. Many studies have suggested that the quality of dietary proteins can be an important modulator of the risks associated with this syndrome [4-7].

In general, plant protein intake is inversely related to the risk of hypertension and cardiovascular disease [4-7]. For example, several studies have shown that dietary soy protein reduces cholesterol and triglyceride (TG) levels and lowers blood pressure in animals and humans [4-9]. Because it has been reported that peptides or protein hydrolysates show greater bioactivities than intact proteins or amino acid mixtures [10,11], bioactive peptides have been produced *in vitro *through chemical or enzymatic hydrolysis of several food proteins, in order to modify and improve the physiological functions of dietary proteins [11,12]. Bioactive peptides are prepared from both plant and animal sources, with the antihypertensive peptides IAP and VY and the hypocholesterolemic peptide IIAEK derived from wheat gliadin, sardine muscle, and bovine milk α-lactoglobulin, respectively [13-17]. For soy protein, several bioactive peptides, including the hypocholesterolemic peptide LPYPR, antihypertensive peptide NWGPLV, antioxidant peptide LLPHH, and anti-obese peptide VRIRLLQRFNKRS, were derived from its major constituents, glycinin and β-conglycinin (BCG) [18-21]. Of the limited number of peptides reported to have hypotrigliceridemic activities, VVYP, VYP, and VTL were identified in hydrolyzed globin from animal blood [22].

In the present study, we investigated the effects of soy protein-derived peptides on hepatic lipid metabolism, especially TG metabolism, both *in vitro *and *in vivo*. We used human hepatoma HepG2 cells, the most suitable and accessible human-derived cells that retain many of the biochemical functions of human liver parenchymal cells [23], for the *in vitro *study. For the *in vivo *study, Otsuka Long-Evans Tokushima fatty (OLETF) rats, which develop a syndrome with multiple metabolic and hormonal disorders that shares many features of human obesity, were used. OLETF rats exhibit hyperphagia, owing to the lack of cholecystokinin receptors; as a result, they become obese and develop hyperlipidemia, fatty liver, and diabetes [24-26].

## Materials and methods

### Preparation of soy peptides

**Figure 1:**
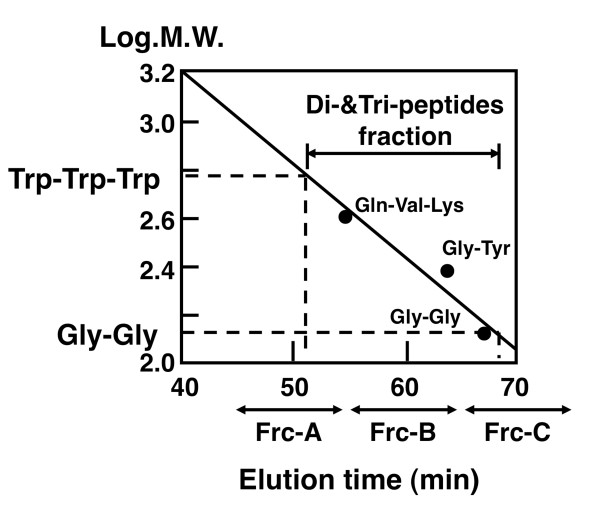
**Calibration of Superdex Peptide HR10/30 gel permeation chromatography column by using standard peptides and the elution profile of the hydrophilic fraction of soy crude peptide-LD3**.

**Figure 2:**
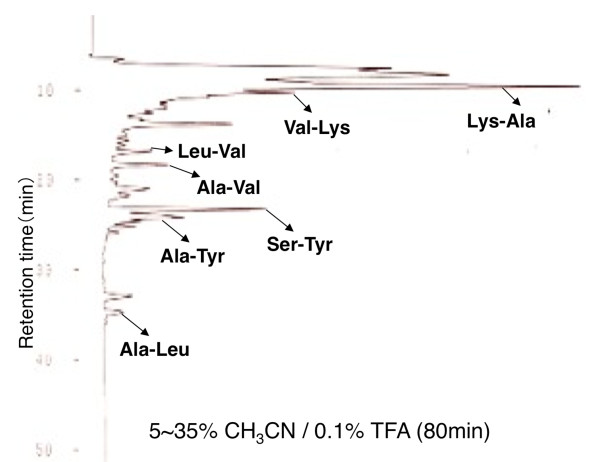
**Reversed phase-high performance liquid chromatography elution profile of 0% CH_3_CN/0.1%TFA fraction peptides on COSMOSIL 5C_18_-AR-II column and identification of di-peptides**.

Preparation of soy crude peptides (SCP)-LD3 was performed by hydrolyze of soy protein isolate with endo-type protease derived from *Bacillus sp*. and fractionation with centrifugation [27]. SCP-LD3 was separated into 2 fractions (hydrophobic fraction and hydrophilic fraction) on the basis of hydrophobicity by using hydrophobic synthetic absorbent (DIAION HP21; Mitsubishi Chemical Corporation, Tokyo, Japan). The amino acid composition of SCP-LD3, hydrophobic fraction, and hydrophilic fraction was analyzed with an automatic amino acid analyzer; results are shown in Table 1. Hydrophilic fraction was further fractionated by gel permeation chromatography-high performance liquid chromatography (GPC-HPLC, Bio-Rad Superdex Peptide HR10/30 column, 30% CH_3_CN/0.1% trifluoroacetic acid (TFA), 0.3 mL/min), and di- or tri-peptide fractions were collected at an elution time ranging from 45 to 75 min (Figure 1; Fraction A, Frc-A, 45-55 min; Fraction B, Frc-B, 55-65 min; Fraction C, Frc-C, 65-75 min). Then, 2 peptide fractions (0% CH_3_CN/0.1% TFA and 20% CH_3_CN/0.1% TFA fractions) were obtained by octadecylsilyl (ODS) open-column chromatography (20 × 60 mm) from Frc-C peptides. Finally, 0% CH_3_CN/0.1% TFA fraction peptides were separated by reversed-phase HPLC (COSMOSIL 5C_18_-AR-II column; Nakalai tesque, Kyoto, Japan), and 7 di-peptides (Figure 2; Ala-Leu, Ala-Tyr, Ala-Val, Leu-Val, Lys-Ala, Ser-Tyr, and Val-Lys) were identified. These di-peptides, except Leu-Val, could be synthesized using a Fmoc solid-phase synthesis method according to the manufacturer's instructions (Kokusan Chemicals, Osaka, Japan). The sequence of the di-peptides was confirmed on a PPSQ-21 amino acid sequencer (Shimadzu Co., Kyoto, Japan).

**Table 1:** Amino acid composition of soy peptides

Amino acid	Soy crude peptide-LD3	Hydrophobic peptide	Hydrophilic peptide
		(%)	
Val	3.78	4.84	4.00
Ile	3.81	5.98	3.15
Leu	8.00	5.41	8.61
Lys	6.20	5.35	7.15
Met	1.50	1.47	1.00
Phe	4.86	8.20	2.80
Thr	4.07	3.45	3.84
Trp	-	-	-
His	2.97	3.05	2.99
Tyr	4.19	4.87	2.32
Cys	1.08	1.50	1.13
Asx	12.11	11.33	12.62
Glx	20.38	13.28	27.14
Pro	5.52	8.23	4.73
Gly	3.82	3.94	4.12
Ser	5.56	4.25	5.52
Ala	4.10	2.94	4.42
Arg	8.11	8.71	7.66

Tryptophan content could not be determined under the present experimental condition.

### Cell culture

HepG2 cells were maintained in Dulbeccos's modified Eagle's medium (DMEM) containing 100 units/mL penicillin and 100 μg/mL streptomycin and supplemented with 10% fetal bovine serum at 37°C in a humidified atmosphere of 5% CO_2_. At approximately 70-80% confluence, the medium was preincubated with 1% bovine serum albumin (BSA)-DMEM with 0.5 mM oleic acid for 24 h. The fatty acid-BSA complex was prepared as described by Van Harken et al [28]. The cellular protein concentration was determined using the method of Lowry et al [29] or the bicinchoninic acid method [30]. To measure cytotoxicity, 3-(4,5-dimethylthiazolyl-2)-2,5-diphenyl tetrazolium bromide (MTT) activity was determined using a MTT Cell Growth Kit (CHEMICON International, Inc., Temecula, CA, U.S.A.); and after 24-h incubation with 1-10 mg/mL of the soy peptides, the MTT activity of HepG2 cells was unaffected in all experiments (data not shown).

### Measurement of cellular TG synthesis

To evaluate the effects of the peptides on cellular TG synthesis, HepG2 cells were preincubated with 1% BSA-DMEM with 0.5 mM oleic acid for 24 h and then incubated with experimental medium (Control: 1% BSA-DMEM; Peptides: 1% BSA-DMEM with 1-10 mg/mL peptides) containing 18.5 KBq [1-^14^C] acetate (American Radiolabeled Chemicals, St. Louis, MO, USA) for 24 h. After incubation, cells were washed once and collected in 2 mL of phosphate-buffered saline using a rubber policeman. Cells were thawed and homogenized with a sonicator (Sonifier 250TM; Branson Ultrasonic Co., CT, USA) before analysis. Total lipids in the cells were extracted and purified by the method of Bligh and Dyer [31], then radioactivities were measured by a liquid scintillation counter (Wallic System 1410; Pharmacia, Uppsala, Sweden). The lipids were fractionated using thin-layer chromatography (TLC) in a solvent mixture of petroleum ether:diethyl ether:acetate (82:18:1, v/v/v). After separation with TLC, the radioactivities of the lipid fractions were quantitated with a bio-imaging analyzer (BAS1000; Fuji Photo Film, Kanagawa, Japan). Activity of TG synthesis was calculated as "cpm per mg protein" and represented as "% of control" in figures.

### Measurement of apolipoprotein B100 secretion

To evaluate the effects of the di-peptides on apolipoprotein B100 (apoB100) secretion, HepG2 cells were preincubated with 1% BSA-DMEM with 0.5 mM oleic acid for 24 h and further incubated with experimental medium (Control: 1% BSA-DMEM; Peptides: 1% BSA-DMEM with 5 mg/mL di-peptides) for 24 h. At the end of the experiment, the media were harvested for the measurement of apoB100 levels, and the cells were used for determination of cellular protein levels. ApoB100 levels in the culture media were quantitated using the ApoB Microwell ELISA Assay Kit (AlerCHEK).

### Animals and diets

All aspects of the animal experiment were conducted according to the guidelines provided by the ethical committee of experimental animal care at Saga University. Male OLETF rats aged 4 weeks were provided by the Tokushima Research Institute (Otsuka Pharmaceutical, Tokushima, Japan). The rats were housed individually in metal cages in a temperature-controlled room (24°C) under a 12-h light/dark cycle. In the first animal experiment, the rats were assigned to 1 of 2 groups (6 rats each), and were fed 1 of 2 diets: (i) a semi-synthetic diet containing (in weight %) casein, 20; corn oil, 7; cornstarch, 15; vitamin mixture (AIN-76™), 1; mineral mixture (AIN-76™), 3.5; DL-methionine, 0.3; choline bitartrate, 0.2; cellulose, 5; and sucrose, 48 (control diet, Con); (ii) a semi-synthetic diet containing (in weight %) SCP-LD3, 19.1; corn oil, 7; cornstarch, 15; vitamin mixture, 1; mineral mixture, 3.5; DL-methionine, 0.3; choline bitartrate, 0.2; cellulose, 5; and sucrose, 48.9 (soy peptide diet, SoyPep) for 2 weeks. In the second animal experiment, the rats were assigned to 1 of 3 groups (6 rats each) and were fed 1 of the following 3 diets: (i) control diet; (ii) soy peptide diet; and (iii) a semi-synthetic diet containing (in weight %) hydrophilic peptide, 11.85; casein, 10; corn oil, 7; cornstarch, 15; vitamin mixture, 1; mineral mixture, 3.5; DL-methionine, 0.3; choline bitartrate, 0.2; cellulose, 5; and sucrose, 46.15 (hydrophilic peptide diet) for 4 weeks. Basal semisynthetic diets were prepared according to recommendations of the AIN-76 [32].

### Measurement of TG levels in serum and liver

All rats were killed by aortic exsanguination under diethyl ether anesthesia, and the liver was excised for analysis. The serum was separated from the blood, and serum TG levels were measured using a commercial enzyme assay kit (Wako Pure Chemicals, Tokyo, Japan). Hepatic lipid was extracted according to the method of Folch et al. [33], and the TG concentration was measured by the method of Fletcher [34].

### Preparation of hepatic subcellular fractions

A piece of liver from each rat was homogenized in 6 volumes of a 0.25 M sucrose solution containing 1 mM ethylenediaminetetraacetic acid in a 10 mM Tris-HCl buffer (pH 7.4). After the nuclear fraction was precipitated, the supernatant was centrifuged at 10,000 × *g *for 10 min at 4°C to obtain mitochondrial fractions. The resulting supernatant was recentrifuged at 125,000 × *g *for 60 min to precipitate microsomes, and the remaining supernatant constituted the cytosol fraction. The protein concentration was determined according to the method of Lowry et al. [29], with BSA used as the standard.

### Assays of hepatic enzyme activity

The enzyme activity of phosphatidate phosphohydrolase (PAP) [35], fatty acid synthase (FAS) [36], malic enzyme [37], glucose 6-phosphate dehydrogenase (G6PDH) [38], and carnitine palmitoyltransferase (CPT) [39] was determined as described elsewhere.

### Statistical analysis

All values are expressed as mean ± SE. Data were analyzed by student *t*-test to assess differences between 2 groups. To assess differences between 3 groups, data were analyzed by 1-way ANOVA, and all differences were analyzed by the Tukey-Kramer post-hoc test (KaleidaGraph, Synergy Software, Reading, PA). Differences were considered significant at *P *< 0.05.

## Results and discussion

The liver is the pivotal organ concerned with lipid metabolism. Nonalcoholic fatty liver disease (NAFLD) is often associated with features of the metabolic syndrome and is emerging as the most common liver disease worldwide [40-42]. NAFLD is the preferred term used to describe the spectrum of liver damage, ranging from hepatic steatosis to steatohepatitis, liver fibrosis, and cirrhosis. Most liver-related morbidity and mortality events are associated with the development of cirrhosis. Cirrhosis is most likely to occur in individuals who have progressed from hepatic steatosis to steatohepatitis. Although the processes through which steatohepatitis evolves from hepatic steatosis are not fully understood, it is necessary to develop effective therapies for the treatment of NAFLD, and the discovery of nutrients that reduce the risk of NAFLD would be useful. We previously discovered novel hypolipidemic dietary components such as functional lipids and phytochemicals by *in vitro *(HepG2 cells) and *in vivo *(OLETF rats) evaluation [43-47].

### Effects of SCP-LD3 on lipid metabolism in HepG2 cells and OLETF rats

**Figure 3:**
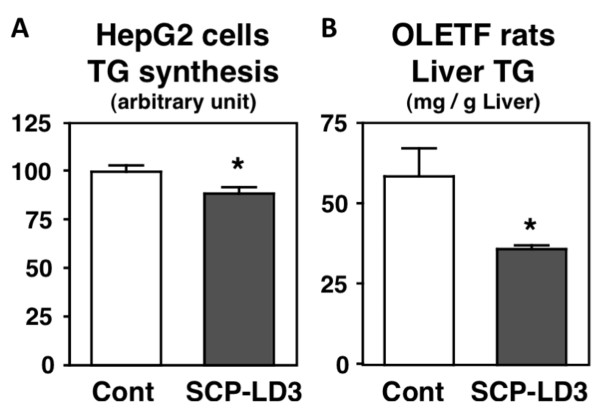
**Effects of soy crude peptide (SCP)-LD3 on triglyceride (TG) synthesis in HepG2 cells (1 mg/mL) and hepatic TG level in Otsuka Long-Evans Tokushima fatty (OLETF) rats**. Values are expressed as mean ± standard error of 5 samples *in vitro *and 6 rats *in vivo*. Asterisks indicate a significant difference at *P *< 0.05.

In the first part of the current study, we evaluated the effects of SCP-LD3 treatment on TG synthesis in HepG2 cells. As shown in Figure 3A, incorporation of [1-^14^C] acetate into the cellular TG fraction was significantly lowered by SCP-LD3 treatment. These results suggest that SCP-LD3 has the ability to normalize lipid abnormalities possibly through the suppression of TG synthesis in hepatocytes. Next, we evaluated the effects of SCP-LD3 on lipid metabolism in OLETF rats. As shown in Figure 3B, 2-week feeding of SCP-LD3 alleviated the obesity-induced TG accumulation in the liver of OLETF rats without significant alterations in other growth parameters such as food intake (control, 29.2 ± 0.6 g/day; SCP-LD3, 29.0 ± 0.6 g/day) and final body weight (control, 539 ± 12 g; SCP-LD3, 531 ± 17 g). Additionally, serum TG level was also lowered by SCP-LD3 feeding in OLETF rats (control, 251 ± 40 mg/dL; SCP-LD3, 143 ± 21 mg/dL, *P *< 0.05). These results indicate that SCP-LD3 has TG-lowering properties, which led us to perform further isolation and identification of hypolipidemic peptide sequences from this peptide during subsequent experiments.

### Effects of hydrophobic and hydrophilic fractions on lipid metabolism in HepG2 cells and OLETF rats

**Figure 4:**
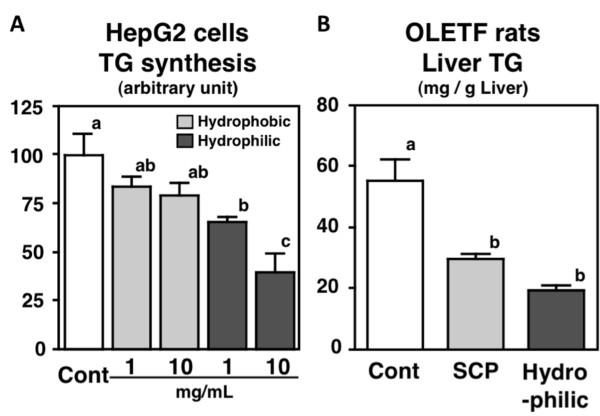
**Effects of soy peptides on triglyceride (TG) synthesis in HepG2 cells (1 or 10 mg/mL) and hepatic TG level in Otsuka Long-Evans Tokushima fatty (OLETF) rats**. Values are expressed as mean ± standard error of 5 samples *in vitro *and 6 rats *in vivo*. Different letters indicate a significant difference at *P *< 0.05.

Several reports have suggested that the amino acid composition of dietary proteins and peptides influences their bioactivities [11]. It has been reported that high amounts of histidine and hydrophobic amino acids contribute to antioxidant potency and that hydrophobic peptides can bind bile acids, thereby enhancing fecal steroid excretion [20,48-50]. In the second part of the current study, we evaluated the effects of hydrophobic and hydrophilic fractions, separated from SCP-LD3 with hydrophobic synthetic absorbent, on TG synthesis in HepG2 cells. As shown in Table 1, the hydrophobic fraction was found to contain a higher amount of phenylalanine, whereas the hydrophilic fraction contained a higher amount of glutamine compared to SCP-LD3. In HepG2 cells, incorporation of [1-^14^C] acetate into the cellular TG fraction was significantly and dose-dependently lowered by hydrophilic fraction treatment (Figure 4A). In the next *in vivo *experiment, we evaluated the effects of the hydrophilic fraction on lipid metabolism in OLETF rats. Though there was no significant alteration in growth parameters (Table 2), feeding of the hydrophilic fraction alleviated hepatomegaly and hepatic TG accumulation in OLETF rats (Table 2, Figure 4B). Moreover, despite the fact that the degree of supplementation with the hydrophilic fraction in the diet (substituted for 10% casein) was half that of SCP-LD3 (substituted for 20% casein), the TG-lowering effects of these 2 diets were almost the same. In agreement with the *in vitro *study, the TG-lowering effects were attributable to the suppression of fatty acid synthesis (represented by lowered activities of FAS, G6PDH, and malic enzyme) and TG synthesis (represented by lowered PAP activity) in the liver of OLETF rats.

**Table 2:** Effects of dietary soy peptides on growth parameters and activities of hepatic triglyceride metabolism-related enzymes in Otsuka Long-Evans Tokushima fatty rats

	Control	Soy crude peptide-LD3	Hydrophilic peptide
Initial body weight (g)	465 ± 11	467 ± 7	463 ± 9
Final body weight (g)	558 ± 10	554 ± 7	550 ± 8
Food intake (g)	743 ± 3	740 ± 8	738 ± 10
Liver weight (g/100 g b.w.)	3.45 ± 0.05^a^	3.14 ± 0.05^b^	3.16 ± 0.06^b^
Hepatic enzyme activity (nmol/min/mg protein)			
FAS	17.0 ± 0.7^a^	13.6 ± 0.8^b^	13.3 ± 1.3^b^
G6PDH	127 ± 7^a^	82.7 ± 4.2^b^	77.0 ± 5.2^b^
Malic enzyme	115 ± 5^a^	79.0 ± 3.7^b^	86.5 ± 2.1^b^
PAP	17.3 ± 1.1^a^	14.5 ± 0.5^b^	14.8 ± 0.5^b^
CPT	5.16 ± 0.31	5.66 ± 0.25	5.42 ± 0.10

FAS, fatty acid synthase; G6PDH, glucose 6-phosphate dehydrogenase; PAP, phosphatidate phosphohydrolase; CPT, carnitine palmitoyltransferase.

Values are expressed as mean ± standard error of 6 rats.

Different superscript letters indicate a significant difference at *P *< 0.05.

### Effects of peptides fractionated by GPC-HPLC on TG synthesis and apoB100 secretion in HepG2 cells

**Figure 5:**
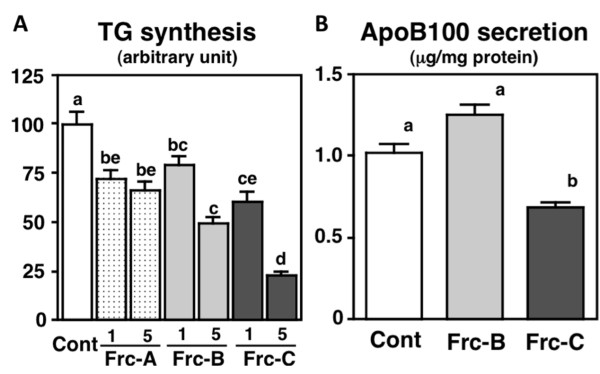
**Effects of fractionated soy peptides (1 or 5 mg/mL) on triglyceride (TG) synthesis and apolipoprotein (Apo) B100 secretion in HepG2 cells**. Values are expressed as mean ± standard error of 5 samples. Different letters indicate a significant difference at *P *< 0.05.

It has been shown that short peptides, mostly di-, tri-, and tetra-peptides, are absorbed more rapidly than free amino acids and that short peptides are absorbed intact by specific peptide transporters such as PepT1 and PepT2 into the blood circulation and transported to target organs [51-54]. Intact absorption of the hypotensive dipeptide Val-Tyr, for example, was confirmed in rats and humans [55-57]. In the third part of the current study, we evaluated the effects of di- or tri-peptide fractions, separated from the hydrophilic fraction with GPC-HPLC (Figure 1), on TG synthesis in HepG2 cells. As shown in Figure 5A, all 3 fractions lowered the incorporation of [1-^14^C] acetate into the cellular TG fraction significantly and dose-dependently. It is known that blood apoB100 level is positively correlated with the incidence of coronary heart disease and that enhanced secretion of apoB100 by the liver is a biomarker of hepatic lipid abnormality [58-60]. Therefore, dietary components that control the rate of apoB100 secretion by the liver are of great interest [61-63]. In the current study, we evaluated the effects of Frc-B and Frc-C treatment on apoB100 secretion from HepG2 cells. Our results show that Frc-C treatment, but not Frc-B treatment, induced a 33% reduction of apoB100 secretion compared with the control medium (Figure 5B).

### Effects of peptides fractionated by ODS column chromatography on TG synthesis and apoB100 secretion in HepG2 cells

**Figure 6:**
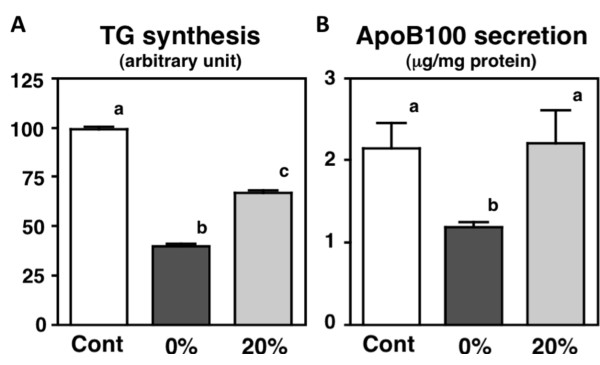
**Effects of fractionated soy peptides (5 mg/mL) on triglyceride (TG) synthesis and apolipoprotein (Apo) B100 secretion in HepG2 cells**. Values are expressed as mean ± standard error of 5 samples. Different letters indicate a significant difference at *P *< 0.05.

In the fourth part of the current study, we evaluated the effects of peptide fractions, separated from the Frc-C peptide with ODS column chromatography, on TG synthesis and apoB100 secretion in HepG2 cells. Our results show that 0% CH_3_CHCN/0.1% TFA fraction lowered both TG synthesis and apoB100 secretion, but 20% CH_3_CHCN/0.1% TFA fraction lowered only TG synthesis, in HepG2 cells (Figure 6A, B).

### Effects of synthesized di-peptides on TG synthesis and apoB100 secretion in HepG2 cells

**Figure 7:**
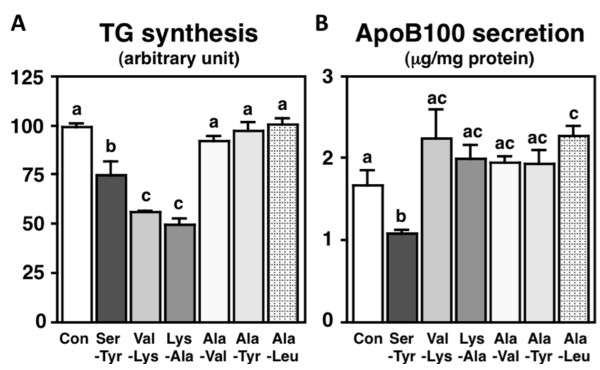
**Effects of synthesized di-peptides (5 mg/mL) on triglyceride (TG) synthesis and apolipoprotein (Apo) B100 secretion in HepG2 cells**. Values are expressed as mean ± standard error of 5 samples. Different letters indicate a significant difference at *P *< 0.05.

Finally, 0% CH_3_CN/0.1% TFA fraction peptides were separated by reverse-phase HPLC, where by 7 di-peptides were identified (Figure 2). Six di-peptides (Ala-Leu, Ala-Tyr, Ala-Val, Lys-Ala, Ser-Tyr, and Val-Lys), but not Leu-Val, could be synthesized, and we evaluated the effects of these di-peptides on TG synthesis and apoB100 secretion in HepG2 cells. We found that 3 di-peptides, Ser-Tyr, Val-Lys, and Lys-Ala, reduced TG synthesis (Figure 7A), and Ser-Tyr additionally reduced apoB secretion (Figure 7B) in HepG2 cells. The presence of 3 di-peptide sequences in the amino acid sequence of soybean components such as glycinin, the BCG alpha subunit (BCG-α), the BCG alpha prime subunit (BCG-α'), the BCG beta subunit (BCG-β), the trypsin inhibitor, and lipoxygenase is shown in Table 3. The presence of the Ser-Tyr sequence is recognized in the amino acid sequence of glycinin, BCG-α, BCG-α', BCG-β, and lipoxygenase. The presence of the Val-Lys sequence is recognized in the amino acid sequence of glycinin, the trypsin inhibitor, and lipoxygenase. The presence of the Lys-Ala sequence is recognized in the amino acid sequence of glycinin, BCG-α, BCG-α', BCG-β, the trypsin inhibitor, and lipoxygenase. Because of the suppressive effects on both lipogenesis and apoB100 secretion in HepG2 cells and the frequent presence in the amino acid sequence of major soybean components, we speculate that Ser-Tyr is the most effective TG-lowering constituent of soy protein isolate-derived peptides.

**Table 3:** Amino acid sequence of major components in soy protein

**Glycinin [Glycine max (soybean)]/CAA37044**
MGKPFTLSLSSLCLLLLSSACFAISSSKLNECQLNNLNALEPDHRVEFEGGLIQTWNSQHPELKCAGVTVSKLTLNRNGLHLP**SY**SPYPRMIIIAQGKGALQCKPGCPETFEEPQEQSNRRGSRSQKQQLQDSHQKIRHFNEGDVLVIPPGVPYWTYNTGDEPVVAISLLDTSNFNNQLDQTPRVFYLAGNPDIEYPETMQQQQQQKSHGGRKQGQHQQEEEEEGGSVLSGFSKHFLAQSFNTNEDIAEKLQSPDDERKQIVTVEGGLSVISPKWQEQQDEDEDEDEDDEDEQIPSHPPRRPSHGKREQDEDEDEDEDKPRPSRPSQGKREQDQDQDEDEDEDEDQPRKSREWRSKKTQPRRPRQEEPRERGCETRNGVEENICTLKLHENIARPSRADFYNP**KA**GRISTLNSLTLPALRQFQLSAQYVVLYKNGIYSPHWNLNANSVIYVTRGQGKVRVVNCQGNAVFDGELRRGQLLVVPQNFVVAEQAGEQGFEYIVFKTHHNAVT**SY**LKDVFRAIPSEVLAH**SY**NLRQSQVSELKYEGNWGPLVNPESQQGSPR**VK**VA
**Beta-conglycinin alpha subunit [Glycine max (soybean)]/BAE46788**
MMRARFPLLLLGLVFLASVSVSFGIAYWEKENPKHNKCLQSCNSERD**SY**RNQACHARCNLLKVEKEECEEGEIPRPRPRPQHPEREPQQPGEKEEDEDEQPRPIPFPRPQPRQEEEHEQREEQEWPRKEEKRGEKGSEEEDEDEDEEQDERQFPFPRPPHQKEERKQEEDEDEEQQRESEESEDSELRRHKNKNPFLFGSNRFETLFKNQYGRIRVLQRFNQRSPQLQNLRDYRILEFNSKPNTLLLPNHADADYLIVILNGTAILSLVNNDDRD**SY**RLQSGDALRVPSGTTYYVVNPDNNENLRLITLAIPVNKPGRFESFFLSSTEAQQ**SY**LQGFSRNILEA**SY**DTKFEEINKVLFSREEGQQQGEQRLQESVIVEISKEQIRALSKRAKSSSRKTISSEDKPFNLRSRDPIYSNKLGKFFEITPEKNPQLRDLDIFLSIVDMNEGALLLPHFNS**KA**IVILVINEGDANIELVGLKEQQQEQQQEEQPLEVRKYRAELSEQDIFVIPAGYPVVVNATSNLNFFAIGINAENNQRNFLAGSQDNVISQIPSQVQELAFPGSAQAVEKLLKNQRE**SY**FVDAQPKKKEEGNKGRKGPLSSILRAFY
**Beta-conglycinin alpha prime subunit [Glycine max (soybean)]/BAB64303**
MMRARFPLLLLGVVFLASVSVSFGIAYWEKQNPSHNKCLRSCNSEKD**SY**RNQACHARCNLLKVEEEEECEEGQIPRPRPQHPERERQQHGEKEEDEGEQPRPFPFPRPRQPRQEGEHEQKEEHEWHRKEEKHGGKGSEEEQDGREHPRPHQPHQKEEEKHEWQHKQEKHQGKESEEEEEDQDEDEEQDKESQESEGSESQREPRRHKNKNPFHFNSKRFQTLFKNQYGHVRVLQRFNKRSQQLQNLRDYRILEFNSKPNTLLLPHHADADYLIVILNGTAILTLVNNDDRD**SY**NLQSGDALRVPAGTTYYVVNPDNDENLRMITLAIPVNKPGRFESFFLSSTQAQQ**SY**LQGFSKNILEA**SY**DTKFEEINKVLFGREEGQQQGEERLQESVIVEISKKQIRELSKRAKSSSRKTISSEDKPFNLRSRDPIYSNKLGKLFEITPEKNPQLRDLDVFLSVVDMNEGALFLPHFNS**KA**IVVLVINEGEANIELVGIKEQQQRQQQEEQPLEVRKYRAELSEQDIFVIPAGYPVVVNATSDLNFFAFGINAENNQRNFLAGSKDNVISQIPSQVQELAFLGSAKDIENLIKSQSE**SY**FVDAQPQQKEEGNKGRKGPLSSILRAFY
**Beta-conglycinin beta subunit [Glycine max (soybean)]/BAB64306**
MMRVRFPLLVLLGTVFLASVCVSLKVREDENNPFYFRSSNSFQTLFENQNGRIRLLQRFNKRSPQLENLRDYRIVQFQSKPNTILLPHHADADFLLFVLSGRAILTLVNNDDRD**SY**NLHPGDAQRIPAGTTYYLVNPHDHQNLKIIKLAIPVNKPSRYDDFFLSSTQAQQ**SY**LQGFSHNILETSFHSEFEEINRVLFGEEEEQRQQEGVIVELSKEQIRQLSRRAKSSSRKTISSEDEPFNLRSRNPIYSNNFGKFFEITPEKNPQPRDLDIFLSSVDINEGALLLPHFNS**KA**IVILVINEGDANIELVGIKEQQQKQKQEEEPLEVQRYRAELSEDDVFVIPAAYPFVVNATSNLNFLAFGINAENNQRNFLAGEKDNVVRQIERQVQELAFPGSAQDVERLLKKQRE**SY**FVDAQPQQKEEGSKGRKGPFPSILGALY
**Trypsin inhibitor [Glycine max (soybean)]/AAF87095**
MPSTWGAAGGGLKLGRTGNSNCPVTVLQDYSEIFRGTP**VK**FSIPGISPGIIFTGTPLEIEFAEKPYCAESS KWVAFVDNEIQ**KA**CVGIGGPEGHPGQQTFSGTFSIQKYKFGYKLVFCITGSGTCLDIGRFDAKNGEGG RRLNLTEHEAFDIVFIEASKVDGIIKSVV
**Lipoxygenase [Glycine max (soybean)]/CAA39604**
MFGIFDKGQKIKGTVVLMPKNVLDFNAITSIGKGGVIDTATGILGQGVSLVGGVIDTATSFLGRNISMQLISATQTDGSGNGKVGKEVYLEKHLPTLPTLGARQDAFSIFFEWDASFGIPGAFYIKNFMTDEFFLVS**VK**LEDIPNHGTIEFVCNSWVYNFR**SY**KKNRIFFVNDTYLPSATPAPLLKYRKEELEVLRGDGTGKRKDFDRIYDYDVYNDLGNPDGGDPRPILGGSSIYPYPRRVRTGRERTRTDPNSEKPGEVYVPRDENFGHLKSSDFLTYGIKSLSHDVIPLFKSAIFQLRVTSSEFESFEDVRSLYEGGIKLPTDILSQISPLPALKEIFRTDGENVLQFPPPHVAKVSKSGWMTDEEFAREVIAGVNPNVIRRLQEFPPKSTLDPTLYGDQTSTITKEQLEINMGGVTVEEALSTQRLFILDYQDAFIPYLTRINSLPTA**KA**YATRTILFLKDDGTLKPLAIELSKPHPDGDNLGPESIVVLPATEGVDSTIWLLA**KA**HVIVNDSGYHQLVSHWLNTHAVMEPFAIATNRHLSVLHPIYKLLYPHYRDTININGLARQSLINADGIIEKSFLPGKYSIEMSSSVYKNWVFTDQALPADL**VK**RGLAIEDPSAPHGLRLVIEDYPYAVDGLEIWDAIKTWVHEYVSLYYPTDAAVQQDTELQAWWKEAVEKGHGDLKEKPWWPKMQTTEDLIQSCSIIVWTASALHAAVNFGQYPYGGLILNRPTLARRFIPAEGTPEYDEM**VK**NPQ**KA**YLRTITPKFETLIDLSVIEILSRHASDEIYLGERETPNWTTDK**KA**LEAFKRFGSKLTGIEGKINARNSDPSLRNRTGPVQLPYTLLHRSSEEGLTFKGIPNSISI

Definition [source]/Accession number. Lys-Ala, KA; Ser-Tyr, SY; Val-Lys, VK.

## Conclusion

**Figure 8:**
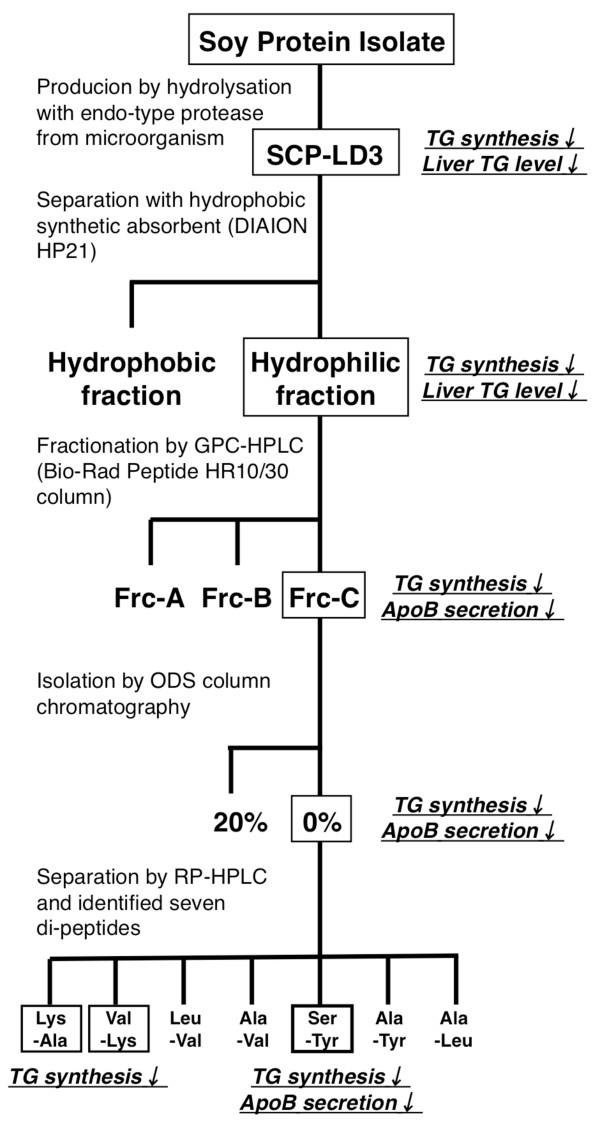
**Screening chart of lipid-lowering di-peptides from soy protein isolate**.

We appear to have isolated novel active peptides with TG-lowering effects from soy protein, including Lys-Ala, Val-Lys, and Ser-Tyr, using *in vitro *and *in vivo *screening systems (Figure 8). The physiological properties of these isolates would be, at least in part, attributable to suppressed lipogenesis in the liver. Further studies are necessary to evaluate the effects of these 3 di-peptides on lipid abnormalities *in vivo *and to determine the lowest effective concentration.

## List of abbreviations

ApoB100: apolipoprotein B100; BCG: β-conglycinin; BSA: bovine serum albumin; CPT: carnitine palmitoyltransferase; DMEM: dulbecco's modified eagle medium; FAS: fatty acid synthase; G6PDH: glucose 6-phosphate dehydrogenase; GPC: gel permeation chromatography; HPLC: high performance liquid chromatography; MTT: 3-(4,5-dimethylthiazolyl-2)-2,5-diphenyl tetrazolium bromide; NAFLD: nonalcoholic fatty liver disease; ODS: octadecylsilyl; OLETF: Otsuka Long-Evans Tokushima fatty; PAP: phosphatidate phosphohydrolase; SCP: soy crude peptide; TFA: trifluoroacetic acid; TG: triglyceride; TLC: thin-layer chromatography.

## Competing interests

The authors declare that they have no competing interests.

## Authors' contributions

NI and KN made substantial contributions to the conception and design of the study, performing the experiment, assembly, analysis and interpretation of data and drafting the manuscript. KS, NY, PERG, SYH, TM and TN participated in experimental work and collection, assembly, analysis of data. HF, KT and TY contributed in planning of the experiment and in discussion of results. All authors read and approved the final manuscript.
